# Non-surgical treatment of hip osteoarthritis. Hip school, with or without the addition of manual therapy, in comparison to a minimal control intervention: Protocol for a three-armed randomized clinical trial

**DOI:** 10.1186/1471-2474-12-88

**Published:** 2011-05-04

**Authors:** Erik Poulsen, Henrik W Christensen, Ewa M Roos, Werner Vach, Søren Overgaard, Jan Hartvigsen

**Affiliations:** 1 Institute of Sports Science and Clinical Biomechanics, University of Southern Denmark, Denmark; 2 Nordic Institute of Chiropractic and Clinical Biomechanics, Odense, Denmark; 3 Institute of Medical Biometry and Medical Informatics, University of Freiburg, Germany; 4 Institute of Clinical Research, University of Southern Denmark, Denmark; 5 Department of Orthopedic Surgery and Traumatology, Odense University Hospital, Denmark

## Abstract

**Background:**

Hip osteoarthritis is a common and chronic condition resulting in pain, functional disability and reduced quality of life. In the early stages of the disease, a combination of non-pharmacological and pharmacological treatment is recommended. There is evidence from several trials that exercise therapy is effective. In addition, single trials suggest that patient education in the form of a hip school is a promising intervention and that manual therapy is superior to exercise.

**Methods/Design:**

This is a randomized clinical trial. Patients with clinical and radiological hip osteoarthritis, 40-80 years of age, and without indication for hip surgery were randomized into 3 groups. The active intervention groups A and B received six weeks of hip school, taught by a physiotherapist, for a total of 5 sessions. In addition, group B received manual therapy consisting of joint manipulation and soft-tissue therapy twice a week for six weeks. Group C received a self-care information leaflet containing advice on "live as usual" and stretching exercises from the hip school. The primary time point for assessing relative effectiveness is at the end of the six weeks intervention period with follow-ups after three and 12 months.

Primary outcome measure is pain measured on an eleven-point numeric rating scale. Secondary outcome measures are the hip dysfunction and osteoarthritis outcome score, patient's global perceived effect, patient specific functional scale, general quality of life and hip range of motion.

**Discussion:**

To our knowledge this is the first randomized clinical trial comparing a patient education program with or without the addition of manual therapy to a minimal intervention for patients with hip osteoarthritis.

**Trial registration:**

ClinicalTrials NCT01039337

## Background

Osteoarthritis (OA) of the hip is a major contributor to pain, decreased physical function and decline in health related quality of life (QoL) [1]. In the western world, it is estimated that 5-11% of the adult population are affected by hip OA, with even higher prevalence rates in the senior population [2-4]. Consequently, an increasing aging population is expected to lead to a steep increase in the number of people affected by hip OA in the coming decades.

Recent international evidence-based guidelines dealing with the management of hip and knee OA recommend a combination of pharmacological and non-pharmacological interventions as first line treatment [5-7]. Initially, patient information and weight reduction are recommended, followed by exercise. If warranted this can be supplemented by pharmacological treatment in the form of paracetamol and/or non-steroid anti-inflammatory drugs (NSAID). In selected patients, when such non-surgical interventions are no longer sufficiently effective, arthroplasty is considered an appropriate treatment option [5].

There is evidence from several trials that exercise therapy is effective [8,9].

Patient education programs have become popular in a range of chronic conditions over the past two decades [10-13]. In the case of OA, authors of a meta-analysis concluded that patients receiving disease specific education in addition to NSAID achieved an added effect of 20% pain reduction when this was compared to NSAID alone [12]. An example of such a disease specific patient information program is the so-called "hip school" which has been shown to be a promising intervention [14]. Fernandes et al have compared the hip school to a group receiving both hip school and supervised exercise therapy and although a small reduction in pain scores was observed within groups at four, 10 or 16 months follow-up, no statistical significant difference was observed between groups at any of the follow-up points [15].

Manual therapy is an umbrella term comprising different manual techniques aimed at decreasing pain and improving function of the musculoskeletal system [16]. Examples include joint manipulation and mobilization, muscular, ligament and capsular stretching and trigger point therapy. Historically, practitioners of manual medicine, chiropractic, physiotherapy and osteopathy have been treating patients with musculoskeletal complaints resulting from OA using these techniques [16-19]. Recently, results from a randomized clinical trial (RCT) showed that patients receiving such manual therapy compared to exercise resulted in more pain reduction, improvement in activities of daily living and general health status after a series of treatments and at 6 months follow-up [20]. There is currently an increased interest in further evaluating the effectiveness of patient education, exercise and manual therapy in this group of patients but few results are available at this time [21-23].

The primary objective of this proof-of-concept study is to assess the effectiveness of hip school with or without the addition of manual therapy in terms of pain severity reduction when compared to a minimal intervention. Second, we will explore if adding manual therapy to the hip school is associated with added benefit.

## Methods

### Study design

Randomized clinical trial

### Participants and recruitment procedure

Patients from primary care with a clinical and radiological diagnosis of hip OA, or with a working diagnosis of clinical hip OA (defined as pain in the groin, buttock or proximal thigh area and restriction on passive hip range of motion) could be referred to the study. General medical practitioners (GPs) and practicing chiropractors from the island of Funen, Denmark were invited to an information meeting about the project followed by an information letter. Subsequently, the principal investigator (EP) personally paid most GP practices on the island a visit, and information about the project was made available on a closed web site for health care professionals by the Region of Southern Denmark. Referrals of eligible patients were made to the Department of Orthopedic Surgery and Traumatology, Odense University Hospital, Denmark either in a written form or by telephone. The referring clinician had the opportunity to hand out a short information leaflet about the study. Eligible patients were then contacted by phone by the principal investigator and asked about symptom location, mode of onset, duration, pain severity, improving and worsening factors, medication use and questions related to the exclusion criteria. If inclusion criteria and none of the exclusion criteria were met, an appointment was made for a clinical examination at the Department of Orthopedic Surgery and Traumatology and a radiographic examination at one of two chiropractic clinics. The radiographic examination included an AP pelvic series and a false profile of the involved hip.

Inclusion and exclusion criteria are listed in table 1.

**Table 1:** Criteria for inclusion and exclusion

Inclusion criteria	Exclusion criteria
- 40-80 years of age	- Other conditions than hip OA appearing to be the cause of the patient's symptoms
- Unilateral hip pain > 3 months	- Bilateral hip pain
- Radiographic measurement of joint space width < 2.00 mm or side difference > 10%	- Indication for hip joint replacement surgery within the next 6 months
- Able to speak and read Danish	- Previous hip or knee joint replacement surgery
	- Hip OA due to hip fracture or infection
	- Rating of worst hip pain during the last week as ≤ 2 on 11-box rating scale
	- Hip dysplasia, Center Edge angle < 25 and Acetabular index Angle > 10
	- Local knee pain originating from the knee on the same side as the hip OA
	- Low back pain dominating over the hip symptoms
	- Inflammatory joint disease
	- Cerebrovascular disease
	- Polyneuropathy or neuromuscular disease
	- Malignant disease
	- Refusal to participate

At the first consultation at the hospital, participants were given time for questions and would sign the informed consent form. An appointment was then scheduled for completion of the baseline self-reported outcome measures and randomization.

**Figure 1:**
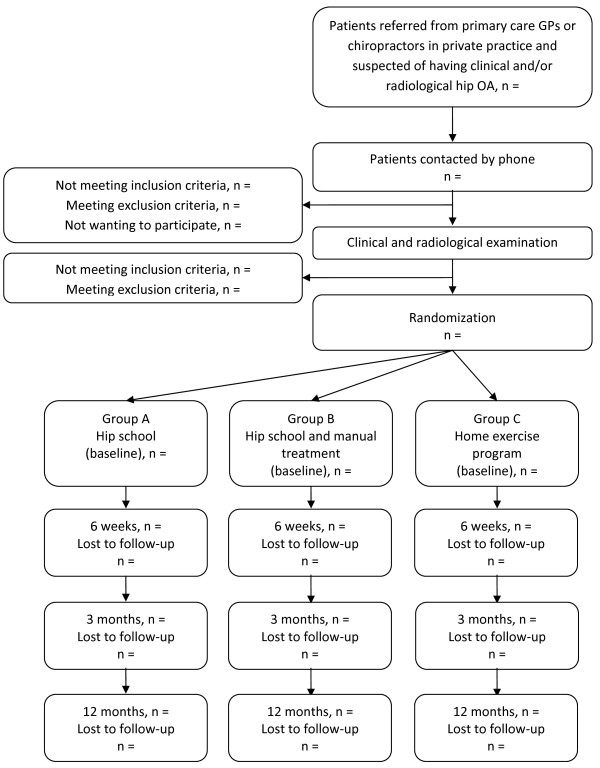
**Study flow chart of randomized clinical trial**.

Figure 1 (study flow chart) illustrates the flow of participants through the study.

### Setting

Initial examination, randomization, manual therapy, and follow-up examinations took place at the outpatient clinic at the Department of Orthopedic Surgery and Traumatology, Odense University Hospital, Denmark. The hip school was taught at the Rehabilitation Unit, Odense University Hospital, Denmark.

Radiographs were taken at one of two pre-selected chiropractic clinics in the town of Odense, Denmark. Clinicians were specially trained for the purpose of this project.

### Randomization

Block randomization was performed using a computer generated list containing a sequence of the letters A - referring to the hip school, B - referring to hip school and manual therapy and C - referring to the minimal information intervention. Block sizes vary with three, six or nine letters. Each letter was written on a piece of paper which was folded and placed in a sealed opaque envelope. The list was generated by a person not involved in the study who is unaware of any information pertaining to the participants. For practical purposes randomization was performed every 8-10 weeks when it was anticipated a sufficient number of patients were referred from primary care to create the two groups involving hip school. The number of envelopes matched the number of patients ready for the randomization and followed the sequential numbers on the generated list. The patient opened the envelope in front of a project nurse who then made an appointment corresponding to the relevant group. The project nurse is not involved in assessment of the patients.

### Interventions

#### Group A: Hip school

The hip school is designed to educate the participants about hip OA. The school was taught over 5 sessions during the 6 weeks intervention period and consisted of one initial personal interview, three group sessions and one follow-up personal session. A specially trained physiotherapist was responsible for teaching the hip school. The content is well described and includes information about epidemiology of hip OA, anatomy of the hip joint and adjacent functional structures, pain distribution and diagnosis of hip OA, recommended activity levels, natural course of the disease, and finally information about treatment options [14]. Stretching exercises for the hip are taught and instructions are given on how to incorporate these into a daily routine. Teaching aids in the form of power point presentations and anatomic models are used. In this study, the original illustrations, with the text translated into Danish (with permission M. Klässbo 03.08.2008), were used.

#### Group B: Hip school and manual therapy

In addition to hip school the patients received manual therapy. The protocol for manual therapy includes a combination of manual soft tissue therapy, stretching and joint manipulation. The soft tissue therapy is trigger point pressure release as described by Travell and Simons [24]. The soft tissue stretching is based on muscle energy techniques as described by Chaitow [25]. The joint manipulation is one of high velocity low amplitude as described by Bergmann, Peterson and Lawrence [26]. The purpose of the manual therapy is to improve elasticity of the muscular, ligamentous and capsular tissue of the hip and posterior joints of the pelvis. Combination of treatment modalities was individualized to each patient according to examination findings at the discretion of the treating clinician. Treatment sessions lasted 15-20 minutes each and treatment was administered twice a week during the six weeks intervention period. The principle investigator EP was the treating clinician in the manual therapy group.

#### Group C: Minimal intervention

A leaflet describing the stretching exercises from the hip school was used as a minimal intervention control. The patients received a short 5 minutes instruction in self-care immediately after randomization by the project nurse and were subsequently handed the exercise leaflet and instructed to incorporate the exercises into their daily routines. Patients were further instructed to live as usual, not to make any changes to use of possible pain medication or initiate other treatment during the following 6 weeks.

The project nurse was making appointments for the participants in group A and B to attend the hip school and for group B the additional manual therapy.

### Period of intervention

Group A and B receiving hip school with or without the addition of manual therapy completed their intervention within a 6 weeks period. All three groups received instructions in a daily stretching program from the hip school and were expected to continue the program after the end of the intervention period.

### Outcome measures

A range of self-reported and clinical outcome measures has been chosen. These include:

#### Primary outcome measure

The CONSORT statement for reporting of randomized trials recommends the use of one primary outcome due to risk of multiplicity of analysis followed by bias in interpretation [27]. The majority of patients in the Hoeksma et al trial rated their main complaint as pain (62% with a mean score of 55.2/100 on a visual analogue scale, standard deviation 22.0) [20]. Therefore we chose pain as the primary outcome.

##### Pain rated on a 0-10 numerical rating scale (NRS)

In hip OA research, OMERACT- OARSI (Outcome Measures in Rheumatology - Osteoarthritis Research Society International) has identified pain as one of three important outcome measure domains [28]. We measure pain using an eleven-point box scale. Patients are asked to rate their "worst" experienced pain during the last week. The eleven-point box scale is a reliable, valid and responsive tool and has been shown to be superior to a visual analogue scale when used in seniors [29].

#### Secondary outcome measures

##### The Hip disability and Osteoarthritis Outcome Score (HOOS)

HOOS is a self-reported patient-relevant outcome measure which includes 5 subscales 1) pain 2) other symptoms 3) function in daily living (ADL) 4) function in sport and recreation and 5) hip related QoL [30]. A 5-point Likert-scale is used and converted into a 100-point scale with zero indicating the worst possible health. The questionnaire and a user's guide, including scoring instructions, are available from http://www.koos.nu.

##### Global assessment of the effect of interventions

The assessment by the patient of a global perceived effect of the treatment is another of the three responder criteria recommended by OMERACT-OARSI [28]. A 7-point Likert-scale is used with the "no change" being the neutral response.

##### Patient Specific Functional Scale (PSFS)

It has been argued that current standardized outcome scores in OA research have the possibility of missing important patient specific disabilities [31]. The PSFS is designed so patients chose up to three activities specifically important to him or her and influenced by their specific condition [32]. An 11-point numerical rating scale is used ranging from "having no problems at all" performing the activity to "is not able to perform the activity".

##### General health status

EuroQoL (EQ-5D) is a self-reported generic general health questionnaire. It includes the following 5 dimensions; mobility, self care, normal activities, pain/discomfort and anxiety/depression and uses a 3-point Likert scale for each dimension [33,34]. It is in this study included for purposes of health economic evaluation and comparison to other hip OA populations.

##### Hip mobility

Correlation has been shown between the amount of radiological defined hip OA and the amount of decline in passive hip range of movement (ROM) [35-37]. Passive hip ROM is defined as the range of movement measured in degrees that an observer is able to move a joint through its full range with no active participation from the patient [26]. Since one of the few studies on the effect of manual therapy has documented a considerable change in ROM following manual therapy, ROM is measured using a standard hand held goniometer [20]. Goniometric measurements of the hip have been examined extensively for reliability in all six ranges of motion and are considered very good (ICC between 0.82 and 0.94 or Pearson correlation coefficients between 0.91 and 0.94) [36,38]. In this study, ROM in extension is not measured for practical reasons, since it requires an assistant to place the goniometer when the patient is lying prone.

##### Use of pain medication

The use of pain medication is recommended as an outcome measure in the most recent evidence-based guidelines for management of hip and knee OA [6]. The patients are asked about type, dosage and frequency.

##### Hip surgery within the follow-up period

Hip surgery within a 12 month follow-up period will be obtained through self-report from the patients. It will be analyzed whether the number of hip surgeries at 12 months follow-up, and the time of surgery, is statistically significantly different between the groups. Hip surgery is defined as total hip arthroplasty.

### Adverse events

Little is known about reactions or adverse events following manual therapy for the extremities or from performing a standardized stretching exercise program. We decided to record occurrence of any reaction or event in the three groups categorized as follows 1) is the reaction related around the hip? 2) mild, moderate or severe 3) when did it start? 4) how long did it last? 5) did it affect activities of daily living? At the end of the 6 week intervention period, a standardized questionnaire was used for each group to record adverse events or reactions to either the hip school, manual therapy or the minimal intervention. The physiotherapist teaching the hip school asked the patient at the last session and completed the questionnaire for participants in group A, the principal investigator administering the manual therapy asked and completed the questionnaire for participants in group B at their last treatment session. A research secretary contacted group C by phone to ask about adverse reaction and completed the questionnaire for them.

### Follow-up

Assessment of the patients is performed at baseline, 6 weeks (after intervention period), 3 and 12 months. The assessments are in the form of self-reported patient questionnaires and a physical examination identical to the baseline examination at all time points. The clinical measurements of the physical examination are performed by the same assessor throughout the study. This assessor is a physical therapist. She is blind to the group allocation of the patients at the time of assessment and is not involved in other parts of the study.

The short term follow-up directly after the 6-weeks intervention is chosen to examine any immediate effect following the manual treatment and the hip school as a maximal effect is anticipated to occur around this time. Any effect due to the high level of interaction with the physiotherapist or chiropractor would further be expected immediately following the intervention period. Three month follow-up is to examine any lasting effect from manual therapy and hip school. Hoeksma et al were able to demonstrate a significant change in pain, hip function and hip range of motion between groups after six months following manual therapy [20]. All three groups are expected to comply with the exercise regimes from the hip school but it is anticipated that the minimal intervention group might not comply at the same level as the other two groups due to lack of continuous positive reinforcement during the intervention period. Twelve months follow-up is important for examining any long term effect. Hip arthroplasty is the ultimate end stage intervention for sufferers of hip OA and it is currently not known if any interventions, pharmacological or non-pharmacological are able to postpone or prevent total hip arthroplasty.

### Blinding

Blinding to treatment allocation (patients, physiotherapist, chiropractor and project nurse involved in the interventions) is not possible due to the nature of the interventions. The assessor analyzing the data (the principle investigator EP) will be blinded as patients are analyzed using recoded identification numbers and group allocation will be unknown when analyzing the data. The recoding will be performed by a person not otherwise involved in the study.

### Statistical analyses

Double data entry will be done by assistants not participating in the study. Baseline characteristics will be described and compared for all 3 groups and between responders and non-responders.

The primary statistical analysis is performed at the end of the 6-week intervention period. This time point was chosen, as we expect the largest treatment effect directly after end of the treatment, and we are interested in a proof-of-principle. The group differences (A vs. C and B vs. C) in pain severity will be analyzed using ANCOVA with adjustment for the baseline values. A significant level of 0.05 will be used. The pre-specified pairwise comparisons between the two active treatments and the self-care control will be analysed using the Dunnett's test and not require the ANCOVA omnibus test to be significant. A post-hoc secondary exploratory analysis of the difference between group A and B will also be performed. The secondary statistical analysis will include the same approach as described above for all the secondary patient rated outcomes. In addition a longitudinal analysis of the primary and secondary patient rated outcomes, incorporating data from baseline, week six, three months and twelve months, will be conducted using a linier mixed model approach [39]. A multiple imputation model will be used for missing data accounting for data missing at random and data missing due to attrition [40,41]. All analyses will follow the intention to treat principle.

Finally, analysis will include calculating the minimal clinically important difference (MCID) based on the change in the primary outcome measure and the patients' global assessment of the overall treatment effect using receiver operating curve (ROC) statistics. Patients' global assessment will be categorized into 1) better 2) no change and 3) worse. The MCID will then be used to estimate how many patients in each group have reached a clinical relevant improvement. Numbers needed to treat (NNT) and odds ratio (OR) for a positive effect of treatment in each group will be presented. All statistical analyses are blinded and will be performed using Stata 10 software (StataCorp, Texas, USA).

### Sample size

For this proof of concept trial, when comparing both A vs. C and B vs. C, we aim to be able to have 80% power (alpha at 0.05) to demonstrate a statistically significant difference of at least 17 percentage points in pain severity at the end of treatment (corresponding to a large effect size of 0.8). Using variability estimates for pain severity from the Hoeksma et al. and other relevant hip osteoarthritis trials as the basis for the sample size calculation, 30 participants in each treatment group are needed, assuming a joint normal distribution for baseline and 6-weeks follow-up with a correlation of 0.3 and equal variances. Allowing for a drop-out of 15% per group, we decided to recruit 106 participants.

### Time line

Inclusion of participants to the main study took place from February 2009 until June 2010. Six weeks and 3 months follow-up have been concluded on all patients. The last group of patients will conclude their 12 months follow-up in June 2011.

### Ethics

Prior to the first hospital appointment, eligible patients received an information package about the study. The package included a thorough explanation of the study, rights when participating in a research project, a copy of the written informed consent form, and directions.

Ethics approval has been granted by the Regional Ethics Committee of Southern Denmark, approval number S-20080027 and the study is registered and approved by the Danish Data Protection Agency, J.nr. 2008-41-1910. The study is registered with clinicaltrials.gov ID NCT01039337 and results will be registered with the same trial register in due time.

## Discussion

To our knowledge this is the first randomized clinical trial comparing the effect of a patient specific education program and manual therapy to a minimal intervention consisting of a simple home stretching exercise regimen in patients diagnosed with unilateral hip OA.

Such a trial is warranted because the vast majority of trials in hip OA until now have been dealing with surgery (73%) or pharmacological interventions (20%) in spite of the fact that recent guidelines for the management of this condition recommend a combination of non-pharmacological and pharmacological interventions as first line treatment [4-7].

The relevance of this research is further based on the continuous increase in the number of patients suffering from OA which is anticipated during the coming decades when both life expectancy and the elder population will rise accordingly [42]. With exploding health care costs and maybe even increased mortality for this patient population the evaluation of simple interventions with proven beneficial effect on functional disability, pain and quality of life should be high on the agenda both from an individual and societal perspective [43,44].

The results so far of the effect of hip school or manual therapy have been based on only a few RCTs [15,20,45] or uncontrolled pre-post comparisons [46,47], and with respect to the direct comparison of manual therapy versus hip school no studies have been performed until now. Our long term interest is in the effect of manual therapy in addition to hip school versus hip school alone, so we included these two groups in our trial. However, for several reasons it is uncertain how well these two therapies will work in our setting: First, the results published so far may reflect publication bias. Second, none of the studies have been performed in Denmark. The hip school does not yet have a tradition in Denmark and manual therapy for hip OA is, to our knowledge, not a common line of intervention. Third, our measurement instruments for success are not identical to those used in the previous studies. Hence we decided to start with a small study assessing the efficacy of the two active treatments. Deciding on a primary outcome in OA research is challenging as it is common for OA patients to rate different domains of the disease experience, e.g. pain and/or reduction in activities of daily living, as their primary complaint. We have decided on pain as the primary outcome which is measured on an eleven-point NRS. At initial contact patients are phoned and asked about possible criteria for exclusion and to avoid a large floor effect on the primary outcome patients are excluded if their pain experience is rated as two or less on the eleven-point NRS.

Strictly speaking the intervention period applies only for the manual therapy and the hip school groups. For the minimal intervention group it is arbitrary because they only receive a one time, five to ten minutes instruction on the exercise program. The purpose of the hip school is to teach the patients an understanding of their condition, to cope with it as well as improve and adapt activities of daily living. This learned effect is of course expected to continue after the intervention period. Any effect of the stretching exercises is expected in all three groups and will not favor any one group over another.

For practical purposes all three groups have a follow-up immediately after the hip school and manual therapy interventions and are then followed at 3 and 12 months to detect changes between groups over time. This does however not reflect common practice as patients with chronic conditions often are followed and treated over months and even years with varying time between consultations depending on symptom severity and flare-ups. Specifically regarding the hip school follow-up sessions would likely encourage modifications of activities of daily living as well as adjustments of exercise regimes. Specifically regarding the manual therapy, it is not uncommon to schedule future consultations for evaluation and treatment if this is perceived beneficial by the chiropractor/therapist and the patient in order to maintain the obtained progress. This practice of follow-up visits is also performed with exercise regimes and patient education programs for reinforcement of confidence and positive behavior as well as adjustments and progression of exercises.

The reasons for inclusion of a control intervention are threefold. First, home programs delivered in the form of a leaflet are often given to this patient group at the time of first diagnosis and our group C thus reflects common practice in many settings. Second, including a control group or minimal intervention group is common in clinical trials that examine the effectiveness of non-pharmacological interventions particularly when long-term follow-up is performed in order to investigate efficiency and cost-effectiveness [48-51]. Third, the effect of a hip school has not previously been compared to a minimal non-pharmacological intervention. It is however possible that patients ending up in the minimal intervention group will feel they are missing out on "treatment" and on their own seek care similar to the hip school or the manual therapy. In order to prevent this, patients in all three groups are encouraged NOT to seek other interventions from baseline to the three months follow-up. This information is provided both in the written material and it is repeated orally at the baseline clinical examination, at randomization and at the 6 weeks clinical examination. Furthermore, patients will be asked specifically at all follow-up points if they have initiated other treatments for their hip condition since the last examination and follow-up.

Any added effect in the group receiving hip school with or without the addition of manual therapy in comparison to the minimal control intervention is of course not necessarily directly linked to the physical component of the interventions. Empathy, social and psychological interactions are important factors in any clinician/therapist patient relationship but the ratio of each to the effect of the physical components is not known. Any measured effect regarding increase in the secondary outcome hip range of motion is however less likely to be influenced by any verbal or empathic contact between patient and clinician/therapist.

The internal validity of the trial is influenced by a positive performance bias by patients participating in the groups receiving hip school with or without the addition of manual therapy whereas patients ending up in the minimal intervention group may experience negative performance bias and feel neglected and not subjected to a "real" treatment. Finally, the list of exclusion criteria may limit the external validity and generalizability, for example, the results may not be directly transferable to patients with hip OA who have a variety of co-morbidities or have hip OA due to moderate or severe hip dysplasia.

Blinding of participants and the providers of the three interventions is another potential problem that may influence the results of the trial. The results from the groups are likely to be influenced by the complex interactions between participants and providers. This includes the verbal communication, physical interaction and empathy between participant and provider. Blinding of the assessor is however possible and this will be done by coding the ID numbers of the participants by a person not involved in the study.

We have designed a study with the main purpose of comparing a patient education program with or without the addition of manual therapy to a minimal intervention in patients with hip OA. The results of this proof of principle study will further inform the design of future RCTs involving non-pharmacological interventions and potentially also the management of patients with early unilateral hip OA.

The results of the study will be submitted to a peer-reviewed journal for publication irrespective of the outcome in accordance with the CONSORT guidelines for reporting of clinical trials [52].

## Competing interests

The authors declare that they have no competing interests.

## Authors' contributions

All authors participated in the design of the study. EP developed the manual treatment protocol, is serving as project manager for the trial and responsible for drafting this paper. JH revised the first draft and JH, HWC, WV, ER and SO commented and revised subsequent drafts. All authors have read and approved the final manuscript.

## Pre-publication history

The pre-publication history for this paper can be accessed here:

http://www.biomedcentral.com/1471-2474/12/88/prepub
